# Supplementary figures and images for: Non-invasive quantification of 18F-florbetaben with total-body EXPLORER PET
Source: EJNMMI Res. 2024 Apr 16;14:39. doi: 10.1186/s13550-024-01104-7 (PMC11021392; doi:10.1186/s13550-024-01104-7)

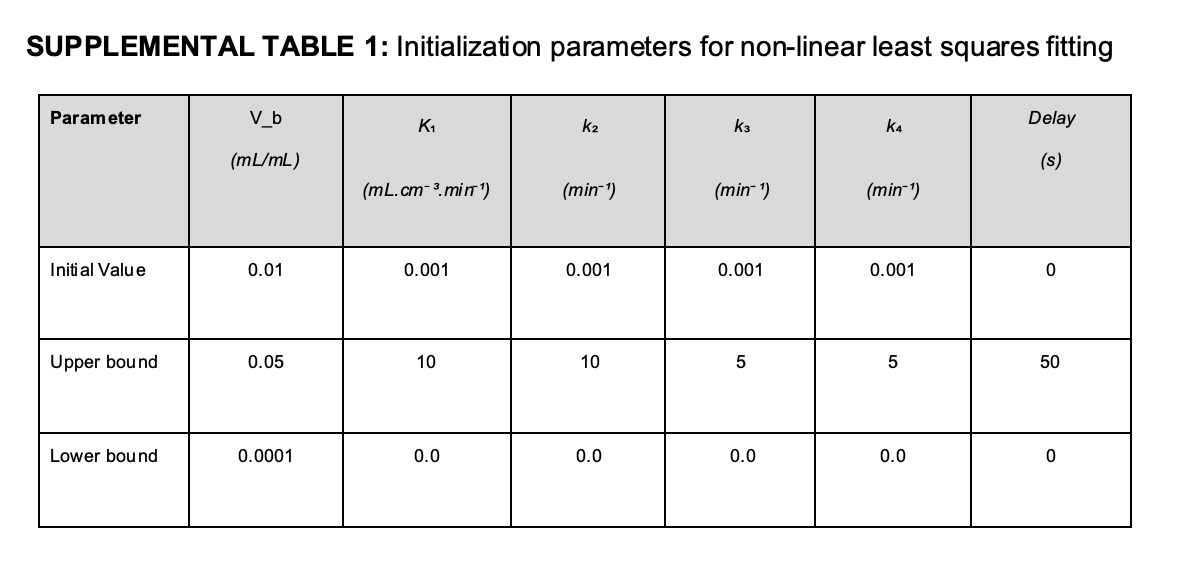

Supplement: Supplementary file 1 — Supplementary Material 1 [file 13550_2024_1104_MOESM1_ESM.png]

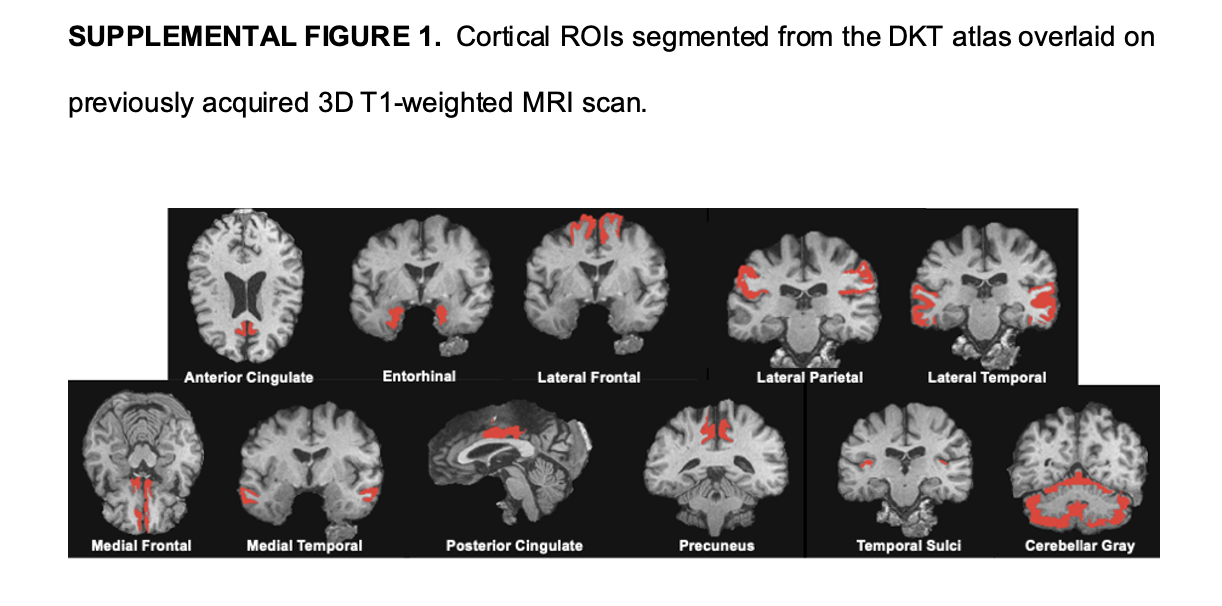

Supplement: Supplementary file 2 — Supplementary Material 2 [file 13550_2024_1104_MOESM2_ESM.png]
