# Supplementary material for: Non-invasive quantification of 18F-florbetaben with total-body EXPLORER PET
Source: EJNMMI Res. 2024 Apr 16;14:39. doi: 10.1186/s13550-024-01104-7 (PMC11021392; doi:10.1186/s13550-024-01104-7)
Supplement: Supplementary file 4 — Supplementary Material 4 [file 13550_2024_1104_MOESM4_ESM.docx]

**SUPPLEMENTAL TABLE 1:** Initialization parameters for non-linear least squares fitting

| **Parameter** | V_b  *(mL/mL)* | *K*₁ *(mL.cm*⁻*³.min*⁻*¹)* | *k*₂  *(min*⁻*¹)* | *k*₃  *(min*⁻*¹)* | *k*₄  *(min*⁻*¹)* | *Delay*  *(s)* |
| --- | --- | --- | --- | --- | --- | --- |
| Initial Value | 0.01 | 0.001 | 0.001 | 0.001 | 0.001 | 0 |
| Upper bound | 0.05 | 10 | 10 | 5 | 5 | 50 |
| Lower bound | 0.0001 | 0.0 | 0.0 | 0.0 | 0.0 | 0 |

**SUPPLEMENTAL FIGURE 1.** Cortical ROIs segmented from the DKT atlas overlaid on previously acquired 3D T1-weighted MRI scan.


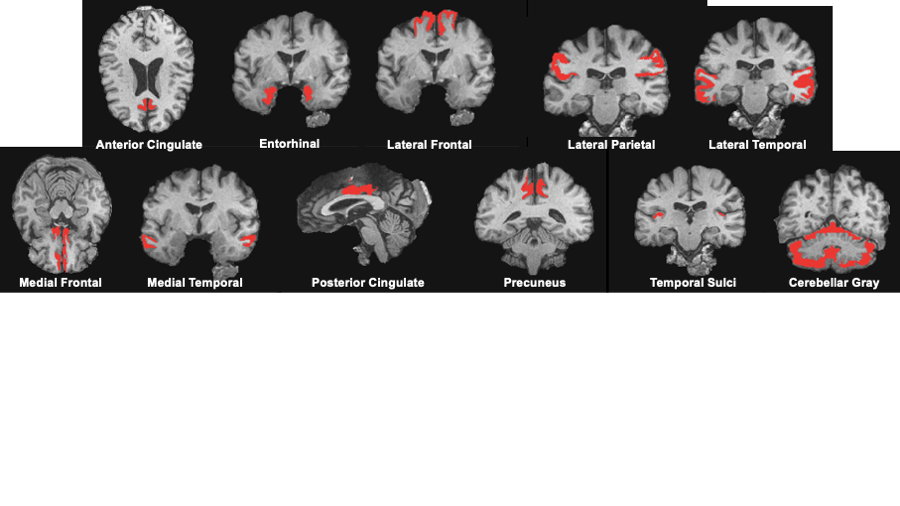


**SUPPLEMENTAL FIGURE 2 .** Linear regression analysis results accounting for subject clustering for Aβ+ (red), Aβ- (black), and all subjects (blue dashed). The following model statistics are for all subjects. (A) V_T_ and SUVR, (r² = 0.46, P<2$e^{-16}$ ). (B) V_S_ and SUVR, (r² = 0.61, P<2$e^{-16}$ ). The following model statistics are for Aβ+ (red) versus Aβ- (black). (A) V_T_ and SUVR, (Aβ+: y = 0.11x -0.31; Aβ-: y = 0.04x +0.61). (B) V_S_ and SUVR, (Aβ+: y = 0.12x +0.36; Aβ-: y = 0.05x + 0.75).

**
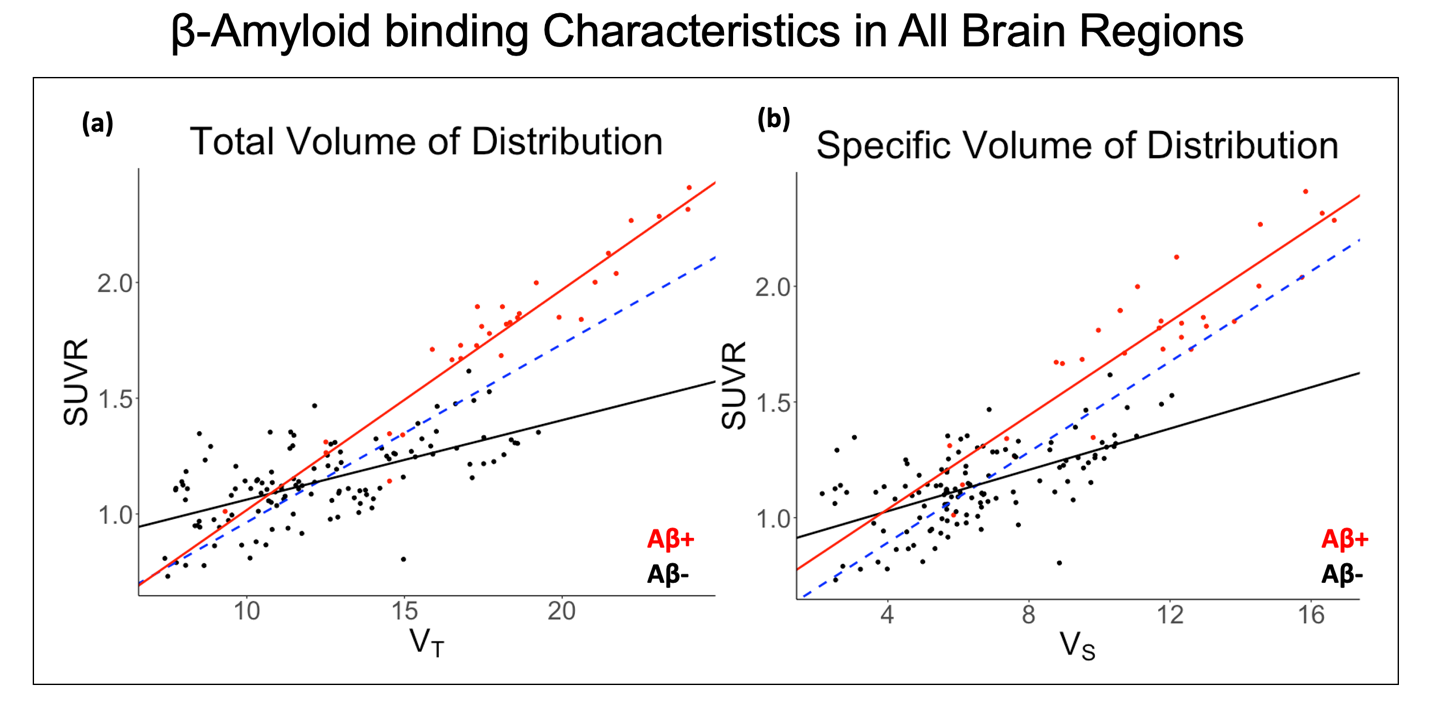
**

**SUPPLEMENTAL FIGURE 3.** Linear regression analysis results accounting for subject clustering for all subjects (blue line). The following model statistics for all subjects SUVR and K1 (r² = 0.05593, P= 0.0022).


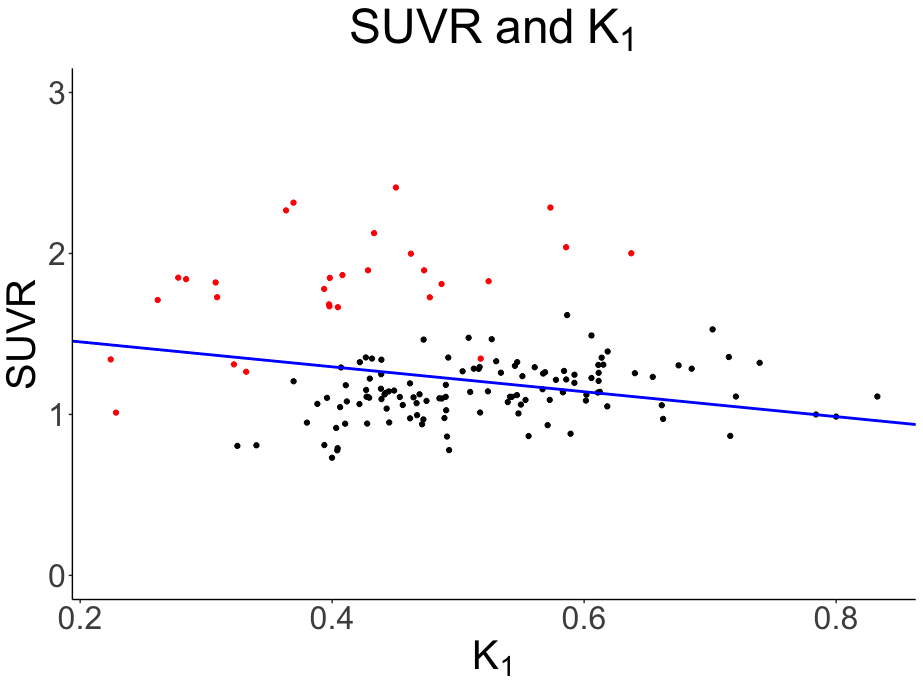


**SUPPLEMENTAL FIGURE 4.** Linear regression analysis results accounting for subject clustering for all subjects (blue line). The following model statistics for all subjects BP_ND_ and K_1_, V_s_ and K_1_, V_T_ and K_1_. **
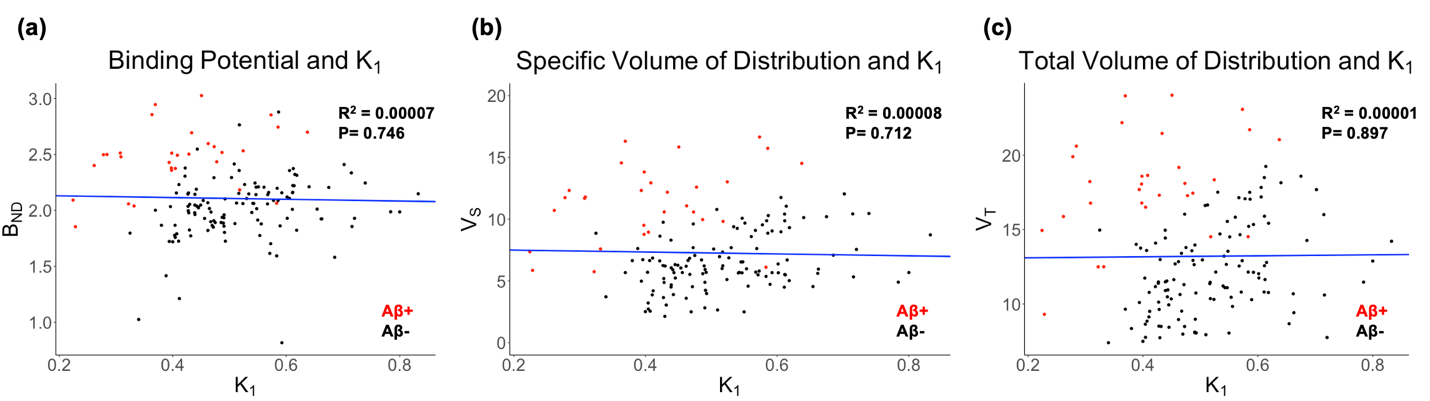
**
