# Supplementary material for: Non-invasive quantification of 18F-florbetaben with total-body EXPLORER PET
Source: EJNMMI Res. 2024 Apr 16;14:39. doi: 10.1186/s13550-024-01104-7 (PMC11021392; doi:10.1186/s13550-024-01104-7)
Supplement: Supplementary file 3 — Supplementary Material 3 [file 13550_2024_1104_MOESM3_ESM.docx]

Reviewer reports:

Reviewer #1: The authors investigate the feasibility of an image-derived input function from aorta artery for amyloid PET quantification using kinetic analysis. Fifteen individuals (3 amyloid positive and 12 amyloid negative) were subjected to a 1010-min dynamic acquisition after 18F-florbetaben injection. Images were analyzed calculating BPND, Vt and Vs. Amyloid positive patients showed increased Vt and Vs compared to amyloid-negative subjects. In addition, these values correlated positively to BPND. The authors conclude that kinetic analysis of 18F-florbetaben using an aortic IDIF is feasible and correlates to BPND values in both, control and amyloid-positive patients. 

The manuscript is well written, the objective is clearly stated, and the methods used adequate for the scientific question. In addition, the results are properly discussed and the conclusion supported by the data. In my opinion, this study is of great interest for the neuroimaging community. The authors can find below some recommendations to improve the manuscript. 

Minor points:
1. P values for the comparisons are only stated for the regressions but not for BPND, Vt and Vs values, only the figures show asterisks. I would recommend to show p values at least for the average values. 

In the revised version of Figure 3, a separate table has been added below the boxplots to denote the original unadjusted p-values. P-values in bold indicate significance when comparing to the false discovery rate (FDR)-corrected threshold values. Linear mixed effects modeling was redone to include the cerebellum (as requested by the second reviewer) for all three parameters (BPND, Vt, and Vs). FDR correction was then applied to account for multiple comparisons. Some significance values have changed but the main take aways from the figure remains the same: (1) Amyloid-positive individuals showed the highest values in all three parameters compared to amyloid-negative cohorts in relation to the interquartile range. (2) Aβ+ group had significantly higher V_T_, and V_S_ derived from kinetic modeling in all index regions, except for the cerebellum (our reference region), entorhinal cortex, lateral parietal, and temporal sulci. (3) BPND seems to show less discriminability and has fewer brain regions that were significantly higher for the Aβ+ group. This may be because BPND was derived from a reference tissue model (MRTM) versus kinetic modeling, however the general trend remains the same as expected. A version of this statement has been added to the discussion to address differences in significance between BP_ND_ and both V_T_/V_S_.

2. Vt and Vs values are correlated to BPND but this does not indicate which of the measurements is better. If the authors have the amyloid values for each subject, a correlation for each value could be made to address which variable reflects better the amyloid load. In addition, usually, amyloid tracers uptake is quantified using SUV. The authors should also compare the values from the kinetic analysis to the SUV to highlight the value of kinetic analysis. 

We agree with the reviewer that amyloid tracers are typically measured with SUV, which should be compared to the kinetic analysis. The original draft included SUVR comparison but was removed for final submission for clarity, since the goal is not to “validate” kinetic measures against SUVR. We have re-added an SUVR comparison discussion along with a supplemental figure. The SUVR information was included as Supplemental Figure 4 to not distract from the main points of the paper demonstrating effective and feasible quantification of amyloid load (see attached figure below) with dynamic kinetic modeling. One of our main points in the discussion is that SUVR has the tendency to overestimate compared to quantitative parameters (e.g., V_T_ and V_S_), which we measured using image-derived input functions. Compared to SUVR, compartment modeling of amyloid enables estimation of total distribution volume without assuming a pseudo-equilibrium, and thus is likely more reliable across variable scan durations and amyloid loads. Because it reflects pseudo-equilibrium of ^18^F-florbetaben as a reversibly binding tracer, SUVR can slightly overestimate model-based values especially for amyloid-positive individuals (Becker GA, Ichise M, Barthel H, et al. PET quantification of 18F-florbetaben binding to β-amyloid deposits in human brains. *J Nucl Med Off Publ Soc Nucl Med*. 2013;54(5):723-731. doi:10.2967/jnumed.112.107185). As shown in Supplemental Figure 2, SUVR reflects increased variability and overestimation for amyloid-positive individuals in red compared to amyloid-negative individuals in black. In comparison, this overestimation is not observed in our kinetic measures as seen in Figure 4.

Amyloid values for blood for CSF were not readily available.

3. The authors discuss that carotid arteries are a worse choice due to partial volume effects. However, there is no comparison in this study. Comparing the same patients using the same kinetic analysis with different IDIFs could give an answer to what weighs more for tracer quantification, the proximity of the carotid to the brain tissue or the effect of partial volume. Ask about depth in main paper for this.

This was a very good point, as we were thinking about this comparison when the study was in its initial stages and placement of the IDIF was being decided. However, we chose not to do a comparison in our patients due to the availability of this comparison on the same EXPLORER system with a different tracer and similar reconstruction parameters. In the study by Li et. al, FDG-EXPLORER PET left ventricle was directly compared to common carotids (Li E, Cherry S, Tarantal A, et al. Identificationand comparisonof image-derived input functions using total-body PET. *J Nucl Med*. 2019;60(supplement 1):520-520.). The common carotids (green) demonstrate much lower peak value and increased tracer delay compared to larger, central vessels (i.e., the aorta) and would therefore lead to inaccurate kinetic parameter results (Figure 1). Even more recent unpublished work with total-body FDG EXPLORER PET further demonstrated that larger blood pools, including ascending aorta (AA) and descending aorta (DA) and well as left ventricle (LV) and right ventricle (RV), are more appropriate for 2TCM kinetic estimate compared to partial volume-limited carotid IDIF (Figure 2). We have adjusted and added statements to the discussion addressing our decision to use the aortic IDIFs based on previous work.

**FIGURE 1**

Li E, et al. 2021

**FIGURE 2**

Li E, et al. 2021

In initial stages of investigation, different size, placement, and shape of IDIF was compared in our subjects to find best blood pool for modeling. All these adjustments withing the descending aorta had no significant difference on modeling results.

4. The discussion is quite long. The paragraph about the uEXPLORER is unnecessarily long. Just discussing that this system allows the implementation of an aortic IDIF should be enough.

This is a great point, we agreed that the section pointed out added extra jargon that was not essential to the message of the paper. Discussion about applications of total-body PET in deep learning and parametric mapping was removed. uEXPLORER is now discussed only for its ability to allow implementation of aorta IDIF and expanding this work to look at different organs. Upon further consideration, the large section regarding the choice of 1TCM versus 2TCM was also removed from the Discussion. We feel it is not necessary as the 2TCM has been the well-established kinetic model for florbetaben (Becker GA, Ichise M, Barthel H, et al. PET quantification of 18F-florbetaben binding to β-amyloid deposits in human brains. *J Nucl Med Off Publ Soc Nucl Med*. 2013;54(5):723-731. doi:10.2967/jnumed.112.107185, Although we did perform 1TCM vs 2TCM kinetic fitting comparisons, it seemed redundant to include this information when other studies have already show 1TCM is not appropriate. Supplemental figure for this discussion portion was also removed.

Reviewer #2: In this paper, 15 subjects received a FBB injection and got a whole body PET scan for 110 minutes.  The goal was to evaluate image derived input function use instead of arterial sampling.  They calculated parameters based on IDIF using 2 tissue compartment and logan graphical analysis.  They also used a reference-region derive quantification using MRTM.  The argument for using IDIF dynamic imaging is that it is independent of perfusion differences across subjects or changes within subjects.  

1. SUVR is currently the industry standard.  The authors' point for needing dynamic imaging with IDIF would be better made if they had compared to SUVR.  It is a shorter scan, which is easier for the subject and scheduling the PET scan.  It is unclear if the authors are suggesting that they intend to start acquiring whole body dynamic FBB scans instead of SUVRs.  They have suggested situations where a whole body dynamic FBB scan would be useful (perfusion differences), but have not shown this.

This was a very good point, as the first reviewer also brought this up. The original draft included SUVR comparison but was removed for final submission for clarity, since the goal is not to “validate” kinetic measures against SUVR. We have re-added an SUVR comparison discussion along with a supplemental figure. The SUVR information was included as Supplemental Figure 4 to not distract from the main points of the paper demonstrating effective and feasible quantification of amyloid load (see attached figure below) with dynamic kinetic modeling. One of our main points in the discussion is that SUVR has the tendency to overestimate compared to quantitative parameters (e.g., V_T_ and V_S_), which we measured using image-derived input functions. Compared to SUVR, compartment modeling of amyloid enables estimation of total distribution volume without assuming a pseudo-equilibrium, and thus is likely more reliable across variable scan durations and amyloid loads. Because it reflects pseudo-equilibrium of ^18^F-florbetaben as a reversibly binding tracer, SUVR can slightly overestimate model-based values especially for amyloid-positive individuals (Becker GA, Ichise M, Barthel H, et al. PET quantification of 18F-florbetaben binding to β-amyloid deposits in human brains. *J Nucl Med Off Publ Soc Nucl Med*. 2013;54(5):723-731. doi:10.2967/jnumed.112.107185). As shown in Supplemental Figure 2, SUVR reflects increased variability and overestimation for amyloid-positive individuals in red compared to amyloid-negative individuals in black. In comparison, this overestimation is not observed in our kinetic measures as seen in Figure 4. We have added statements in the Discussion to discuss SUVR versus dynamic scanning in our study; quantification with kinetic modeling can achieve multiple kinetic parameters and reduce overestimation bias in SUVR, which is a limitation of SUVR despite its shorter scan time. In addition to perfusion, we also aim to continue acquiring dynamic scans for research purposes to investigate amyloid signal and links across different organs in the body, and this has also been added to the Discussion.

We have added a new figure showing perfusion differences between the two amyloid status groups. Whole body dynamic ^18^F-florbetaben PET can give insight to possible perfusion differences in amyloid positive and amyloid negative individuals. Linear mixed effects modeling was done to consider differences in K_1_ between the two groups. FDR correction was applied to accounts for multiple comparisons. Only the entorhinal cortex and temporal sulci had significantly lower perfusion in the amyloid positive compared to amyloid negative. Perfusion was lower in all other brain regions in amyloid positive group but was not significant. Additionally, when considering a composite of all brain index regions there was a significant difference in perfusion between amyloid positive and amyloid negative groups (P<0.05, P=0.0283).

2. They have suggested that a population-based metabolite correction is sufficient, but they don't discuss variability in metabolite correction.

We have added a section to the discussion to address this point: Variability across individuals may be present in metabolite correction particularly at later time points, due to fast metabolism of 18F-labeled amyloid tracers, which can lead to partially inaccurate extraction fractions from kinetic modeling (Nelissen N, Laere KV, Thurfjell L, et al. Phase 1 Study of the Pittsburgh Compound B Derivative 18F-Flutemetamol in Healthy Volunteers and Patients with Probable Alzheimer Disease. *J Nucl Med*. 2009;50(8):1251-1259. doi:10.2967/jnumed.109.063305). Variability due to metabolite correction can be further exaggerated as blood delivery varies between subjects and disease group, especially since aging and AD is linked to changes hepatic function and therefore the amount of radiometabolites (Zanotti-Fregonara P, Chen K, Liow JS, Fujita M, Innis RB. Image-derived input function for brain PET studies: many challenges and few opportunities. *J Cereb Blood Flow Metab*. 2011;31(10):1986-1998. doi:10.1038/jcbfm.2011.107). On the contrary, Patt et al., found no significant difference between AD patients and healthy controls for both 18F-florbetaben metabolic profile, suggesting that applying the same metabolite fraction to both healthy and AD population, as done in our study, may still result in relatively low variability (Patt M, Schildan A, Barthel H, et al. Metabolite analysis of [18F]Florbetaben (BAY 94-9172) in human subjects: a substudy within a proof of mechanism clinical trial. *J Radioanal Nucl Chem*. 2010;284(3):557-562. doi:10.1007/s10967-010-0514-8). Nonetheless, applying a single population-based metabolite correction to two different populations may introduce additional statistical variability in our parameter estimates (Zanotti-Fregonara P, Chen K, Liow JS, Fujita M, Innis RB. Image-derived input function for brain PET studies: many challenges and few opportunities. *J Cereb Blood Flow Metab*. 2011;31(10):1986-1998. doi:10.1038/jcbfm.2011.107).

3. It seems they should have included a comparison with SUVR quantification to make their point about the need for a 110min scan (over 5x longer than standard SUVR scan) on a whole body PET/CT which is most likely 5x more expensive than most PET/CTs.

This was a very helpful comment, and in fact it ties into the reviewer’s point #1 above. We have added a supplemental figure that shows this comparison (reproduced below) and text in discussion section to address this point. There are definitely cases for which simple SUVR calculations are most appropriate, such as routine clinic practice. However, dynamic scanning can provide more insight into amyloid load as it reflects many different tracer parameters (perfusion, extraction fraction, blood flow) and considers physiological variables that SUVR does not.

4. They could show the impact of perfusion on adding noise (perfusion-related?) to the SUVR quantification that was not present in the 2TCM or Logan DVR quantification.

K1 added noise to SUVR quantification that was not observed in the 2TCM or Logan analysis. Higher variability was observed in SUVR quantification compared to the other two measures (SUVR R^2^ = 0.056, V_T_ R^2^ = 0.00001, Logan_V_T_ R^2^ = 0.00089). This was added to the methods section and discussed further in the discussion section. (further described in point 12).

5. Ideally, they would have acquired arterial sampling on a few of the subjects and actually calculated the difference between the two, but obviously that is not feasible now that the data is collected.  

This is a very good point, as it was also brought up many times in the presentation of this work at conferences. Unfortunately, as the reviewer has pointed out, it is no longer feasible since the data has already been collected. Arterial sampling was not in the scope of the project during its initial stages of creation. However, we have added a statement: “In addition, directly comparing acquired arterial blood sampling with an IDIF could have further supported IDIF-based kinetics as a non-invasive alternative but was not within the scope of the study.” to the limitations to highlight this and draw attention for the need or arterial sampling is similar future studies to make a better direct comparison of IDIF against arterial sampling.

6. Is it true that Florbetaben has more specific binding and less off target binding than PIB?  What is the affinity and how does that compare to other amyloid tracers?

This question was very helpful when reviewing the confusion in the statement originally made in the paper. We have adjusted and clarified that we were not referencing PIB specifically as a first-generation tracer, but rather older tracers such as ¹⁸F-DDNP. Florbetaben affinity and binding is relatively like other current amyloid tracers, however the main difference lies in the tracer half-life. Another statement discussing the advantages of the longer half-life has been included. Added sentence here: Current amyloid tracers such as ¹⁸F-florbetaben, a second-generation amyloid tracer with more specific binding and less off-target binding relative to the first generations, (such as¹⁸F-DDNP). Although similar binding affinity and kinetic performance is observed in [C-11]PiB and ¹⁸F-florbetaben, the half lives are 20 minutes and 110 minutes respectively demonstrating one advantage of ¹⁸F such that longer half lives allow for greater distribution radius therefore increasing its availability (Delrieu J, Bateman RJ, Touchon J, Sabbagh M, Cummings J. The Future of AD Clinical Trials with the Advent of Anti-Amyloid Therapies: An CTAD Task Force Report. *J Prev Alzheimers Dis*. 2022;9(3):393-399. doi:10.14283/jpad.2022.48,Sabri O, Seibyl J, Rowe C, Barthel H. Beta-amyloid imaging with florbetaben. *Clin Transl Imaging*. 2015;3(1):13-26. doi:10.1007/s40336-015-0102-6).

7 In introduction, you say BPND is less sensitive to cerebral blood flow changes and "noise related to SUVR."  Be more specific about other sources of noise related to SUVR quantification. Clarify if enough detail

We have elaborated on other sources of noise related to SUVR quantification which arise from tracer clearance and/or extraction fraction. Added sentence here: BP_ND_ measurements from dynamic PET also benefit from being less sensitive to changes in cerebral blood flow (CBF) and noise related to SUVR such as tracer clearance and/or extraction fraction (Heeman F, Yaqub M, Lopes Alves I, et al. Simulating the effect of cerebral blood flow changes on regional quantification of [18F]flutemetamol and [18F]florbetaben studies. *J Cereb Blood Flow Metab*. 2021;41(3):579-589. doi:10.1177/0271678X20918029)

8. Methods: After equations 1 and 2, you say, 'Vb represents blood vlume fraction" instead of "volume"

We have fixed this typo in the manuscript, it now says volume.

9. Equation 5, describe VND

Added a description of VND to methods. Added sentence here: *V*_ND_ represents the distribution volume of nondisplaceable compartment relative to total concentration of tracer in plasma.

10. Figure 3: report the BPND, VT and VS in the reference region (cerebellar gray) used for MRTM.   

The BP_ND_, V_T_, and V_S_ is now reported in figure 3 for the cerebellum. In the revised version of Figure 3, a separate table has been added below the boxplots to denote the original unadjusted p-values (as requested by the other reviewer). P-values in bold indicate significance when comparing to the false discovery rate (FDR)-corrected threshold values. Linear mixed effects modeling was redone to include the cerebellum (as requested by the second reviewer) for all three parameters (BPND, Vt, and Vs). FDR correction was then applied to account for multiple comparisons. Some significance values have changed but the main take aways from the figure remains the same: (1) Amyloid-positive individuals showed the highest values in all three parameters compared to amyloid-negative cohorts in relation to the interquartile range. (2) Aβ+ group had significantly higher V_T_, and V_S_ derived from kinetic modeling in all index regions, except for the cerebellum (our reference region), entorhinal cortex, lateral parietal, and temporal sulci. (3) BPND seems to show less discriminability and has fewer brain regions that were significantly higher for the Aβ+ group. This may be because BPND was derived from a reference tissue model (MRTM) versus kinetic modeling, however the general trend remains the same as expected. A version of this statement has been added to the discussion to address differences in significance between BP_ND_ and both V_T_/V_S_.

11. Table 1: focusing on k3 and k4 for amyloid positive and amyloid negative subjects.  It was my understanding that k3 signifies the rate constant for tracer to the specifically bound region, and k4 was the off-rate for the specifically bound region.  Therefore k3/k4 could be an estimate for the amount of tracer that was specifically bound (the SRTM estimate for BPND).  In your table, the amyloid negatives would have a higher k3/k4 than amyloid positives, this seems wrong.

This comment was very helpful and caused us to reevaluate the data. In Table 1, we only focus on 3 out of the 10 total brain index regions and the reference region. The rate parameter data for amyloid positive and amyloid negative was incorrectly inputted into table. All other values in the table were input correctly. Upon reevaluating and recalculating the average values we find average extraction fraction (k_3_/k_4_) for the 3 brain index regions listed here to be for amyloid positive (1.8066) and for amyloid negative (0.9855), which agrees with your statement. Average values across all brain regions show similar values for both groups. These values are an average across all index regions and exclude the cerebellar reference region: amyloid positive (1.781) and amyloid negative (1.523). Thank you for pointing our attention to this typo.

12. Discussion - say that this would be a good way to model change in amyloid in someone who has reduced perfusion.  This is not shown, though.  It would be useful if there had been correlations between K1 and SUVRs (hopefully there would be a significance where there was no significance where there was no significance with BPND, VS or VT).

Related to the reviewer’s point #1, we have added models of perfusion across the groups to the supplemental section and added commentary in the discussion. (See point 1 above). We observed no significance between K_1_ with BP_ND_, V_T_, Logan V_T_, or V_S_, but significance was observed with SUVR. In contrast, we observed a negative correlation of K_1_ with SUVR (figures reproduced below). This could be because perfusion is adding noise and biological variation to SUVR quantification compared to the modeling techniques.

13. In discussion, review the variability of metabolite correction across subjects, what causes that variability, is it correlated with age, and posit the impact on using a population-based metabolite correction would have.

We have added the following statement: Variability across individuals may be present in metabolite correction particularly at later time points, due to fast metabolism of 18F-labeled amyloid tracers, which can lead to partially inaccurate extraction fractions from kinetic modeling (Nelissen N, Laere KV, Thurfjell L, et al. Phase 1 Study of the Pittsburgh Compound B Derivative 18F-Flutemetamol in Healthy Volunteers and Patients with Probable Alzheimer Disease. *J Nucl Med*. 2009;50(8):1251-1259. doi:10.2967/jnumed.109.063305). Variability due to metabolite correction can be further exaggerated as blood delivery varies between subjects and disease group, especially since aging and AD is linked to changes hepatic function and therefore the amount of radiometabolites (Zanotti-Fregonara P, Chen K, Liow JS, Fujita M, Innis RB. Image-derived input function for brain PET studies: many challenges and few opportunities. *J Cereb Blood Flow Metab*. 2011;31(10):1986-1998. doi:10.1038/jcbfm.2011.107). On the contrary, Patt et al., found no significant difference between AD patients and healthy controls for both 18F-florbetaben metabolic profile, suggesting that applying the same metabolite fraction to both healthy and AD population, as done in our study, may still result in relatively low variability (Patt M, Schildan A, Barthel H, et al. Metabolite analysis of [18F]Florbetaben (BAY 94-9172) in human subjects: a substudy within a proof of mechanism clinical trial. *J Radioanal Nucl Chem*. 2010;284(3):557-562. doi:10.1007/s10967-010-0514-8). Nonetheless, applying a single population-based metabolite correction to two different populations may introduce additional statistical variability in our parameter estimates (Zanotti-Fregonara P, Chen K, Liow JS, Fujita M, Innis RB. Image-derived input function for brain PET studies: many challenges and few opportunities. *J Cereb Blood Flow Metab*. 2011;31(10):1986-1998. doi:10.1038/jcbfm.2011.107). In our data, difference in peak values in before and after metabolite correction had an average value of 4.67Kbqcc and standard deviation of 1.4 Kbqcc, revealing that there is some variability in this correction applied to our data. No significant correlation was observed between peak difference and age (P=0.4; y = -0.05x + 8.5)
